# Development and evaluation of a novel vaccine against prevalent invasive multi-drug resistant strains of *Streptococcus pneumoniae*

**DOI:** 10.7717/peerj.2737

**Published:** 2016-11-30

**Authors:** Rehab H. Bahy, Hayam M. Hamouda, Amal S. Shahat, Aymen S. Yassin, Magdy A. Amin

**Affiliations:** 1 Department of Microbiology, National Organization for Drug Control and Research, Cairo, Egypt; 2 Department of Biochemistry, Basic Medicinal Science, National Organization for Drug Control and Research, Cairo, Egypt; 3 Department of Microbiology and Immunology, Faculty of Pharmacy, Cairo University, Cairo, Egypt

**Keywords:** *S. pneumoniae*, Serotyping, Capsular polysaccharide, Vaccine

## Abstract

*Streptococcus pneumoniae* is a pathogen that causes serious invasive infections, such as septicemia, meningitis and pneumonia in addition to mild upper respiratory tract infections. Protection from pneumococcal diseases is thought to be mediated mainly by serotype-specific antibodies to capsular antigens. Pneumococcal conjugate vaccine consists of sugars (polysaccharides) from the capsule of the bacterium *S. pneumoniae* that are conjugated to a carrier protein. Three pneumococcal conjugated vaccines, each directed against a group of serotypes, are registered in Egypt; however, local vaccine production is required to cover the most prevalent serotypes. In this work, capsular polysaccharide from the most current and prevalent serotypes in Egypt were extracted, purified and conjugated to bovine serum albumin (BSA). The polysaccharide protein conjugate was purified through ultrafiltration technique and molecular size distribution was compared to an available vaccine. The immunogenicity of the prepared vaccine was examined via two methods: First, by measuring the levels of the elicited antibodies in the sera of the vaccinated mice; second, by challenging the vaccinated groups of mice with approximately 10^7^ CFU of each specific serotype and determining the degree of protection the developled vaccine offers. Our results show that the developed conjugated capsular polysaccharide vaccine is highly immunogenic and protective in mice. This finding illustrates the importance of tracking the most recent and predominant peneumococcal serotypes to generate effective vaccines, instead of using expensive imported vaccines with large number of serotypes which might not be even present in the community.

## Introduction

*Streptococcus pneumoniae*, most commonly known as pneumococcus, is a pathogen that causes serious invasive infections, such as septicemia, meningitis and pneumonia as well as mild upper respiratory infections. Pneumococcus is the sixth most frequently isolated organism from human patients (Harakeh, Yassine & El-Fadel, 2006). It has one of the largest public health and economic impacts among bacterial infectious disease agents in both developing and industrialized countries. Pneumonia kills more children than any other illness, more than AIDS, malaria and measles combined (Wardlaw, Johansson & Hodge, 2006). Approximately, 2.6 million children <5 years of age die annually of pneumonia predominantly in the developing world. According to the UNICEF, in Egypt only half of the children with pneumonia are taken to an appropriate healthcare provider, and less than 20% receive antibiotics (UNICEF, 2013). Previous studies performerd to investigate the prevalent serotypes distribution in Egypt indicated that, the serotypes 6B, 1, 19A, 23F and 6A were the most prevalent. (Afifi et al., 2007; Wasfy et al., 2005).

Protection from pneumococcal disease is thought to be mediated mainly by serotype-specific antibodies to capsular antigens (AlonsoDeVelasco et al., 1995). Over the years, two kinds of vaccine have been developed: pneumococcal polysaccharide vaccine (PPV) and pneumococcal conjugate vaccine (PCV) (Williams & Masterton, 2008). Pneumococcal conjugate vaccine consists of sugars (polysaccharides) from the capsule of the bacterium *S. pneumoniae* that are conjugated to a carrier protein. Unlike the pneumococcal polysaccharide vaccine, the pneumococcal conjugate vaccine protects children younger than two years of age. It protects against severe forms of pneumococcal disease, such as pneumonia, meningitis and bacteremia. Three pneumococcal conjugated vaccines are currently registered in Egypt and all of them carry certain fixed multivalent serotypes regardless of the types that are prevalent: PCV7, with serotypes: (4, 6B, 9V, 14, 18C, 19F and 23F). PCV10, with serotypes (1, 4, 5, 6B, 7F, 9V, 14, 18C, 19F and 23), and PCV13, which includes serotypes (1, 3, 4, 5, 6A, 6B, 7F, 9V, 14, 18C, 19A, 19F and 23F).

In our recent work, it was found that currently the most predominant serotypes in Egypt are serotypes 6A/B and 19F of invasive *S. pneumoniae* which also showed alarming levels of multi drug resistance (Bahy et al., 2016). Both serotypes all together accounted for over 50% of the prevalent types. The goal of this study is to develop a pneumococcal capsular polysaccharide conjugated vaccine against these current major prevalent serotypes of *S. pneumoniae* and to assess its immunoginic effect on animals models. The efficacy of the prepared vaccine can be used as a base for production of local pneumococcal polysaccharide vaccine which would alleviate the need to import costly vaccines with multivalent serotypes, with an imposed risk of added toxicity and which might not be relevant to the prevalent ones.

## Materials and Methods

### Statement of ethical approval

All the experiments involving animals, in this study, were approved by the the Research Ethics Committee (REC) of the Faculty of Pharmacy, Cairo University, Cairo, Egypt with approval number MI (492).

### Extraction and purification of capsular polysaccharide (CPS) from *S. pneumoniae*

CPS was prepared from *S. pneumoniae* strain (6A/B or 19F) in one-liter flasks containing 500 ml of brain heart infusion broth and incubated at 37 °C for 24 h, growth was stopped by adding formaldehyde to a final concentration of 0.2% (wt/vol), cells were separated by centrifugation at 4 °C for 30 min at 730 × g, cells were lysed with sodium deoxycholate (0.1%, wt/vol).The mixture was centrifuged for 15 min at 13,800 × g at 4 °C, the supernatant was collected and ethanol was added to a final concentration of 25%. Then the mixture was centrifuged for 2 h at 13,800 × g at 4 °C and the supernatant was collected.

For purification of capsular polysaccharide from nucleic acids, the supernatant obtained above was centrifuged for 5 min at 730 × g at 4 °C. The sediment was collected and re-suspended in Tris-MgSO_4_ buffer at one-fourth the volume originally used to extract the paste. Then, 1.5 mg deoxyribonuclease I, and 0.75 mg ribonuclease-A per 100 gm of original wet paste were added and incubated for 18 h at 37 °C.

For the purification of the capsular polysaccharide from proteins, an equal volume of phenol-acetate solution was added to the nuclease-treated prep, shaken for 30 min (4 °C), centrifuged for 15 min at 18,300 × g and the aqueous phase was collected as the purified capsular polysaccharide (Mallick, 2009).

### Polysaccharides identification using gas chromatography–mass selective detector (GC–MSD)

#### Methanolysis

Purified pneumococcal polysaccharides were dried under vacuum using rotary evaporator at 50 °C. To each tube, methanol was added and the extract was evaporated to dryness using rotary evaporator at 50 °C.

#### Derivatization

A total of 1 ml of the pure compound methanolic solution was added into a 1-ml screw-topped vial and evaporated under a stream of nitrogen at 40 °C to dryness. Two ml of 1N HCl were added and the solution was hydrolyzed at 100 °C for 6 h in an oven. The solution was evaporated to dryness at 40 °C under stream of nitrogen. After that, 0.5 ml of isopropanol (HPLC grade) was added to remove any residue of water, shaken gently, and evaporated to dryness under a stream of nitrogen at 40 °C. An aliquot of 250 µl of oximation solution (2.5% of hydroxyl amine hydrochloride in anhydrous pyridine) was added and put in an oven at 80 °C for half an hour. After cooling, 0.5 ml of sialylation reagent (trimethylchlorosilane (TMS): N, N-O bis-(trimethylsilyl) acetamide ,1:5 by volume) was added and put in an oven at 80 °C for 30 min.

#### GC–MSD

GC/MS was performed in Agriculture Research Center, Cairo, Egypt by Agilest model 5890 GC interfaced to 5972 mass selective detector. HP-5MS capillary columns (30 m × 0.25 mm × 0.25 µm film thickness) were used for GC. Helium was used as the carrier gas at a constant flow rate of 1.5 ml/min. The oven conditions included an initial temperature of 80 °C and an initial time of 1 min, 10 °C/min to 10 °C, and finally 6 °C/min to 300 °C. The inlet temperature was kept constant at 220 °C (Ronald & Ronald, 1991).

#### HPLC analysis of sugars

Samples of serotypes 19F, 6A/B and the commercial product Synflorix (10 serotypes) were filtered through a 0.45 µm membrane. Analysis of the polysaccharides in the filtrate was performed using HPLC, Shimadzu Class-VPV 5.03 equipped with refractive index RID-10A Shimadzu detector, LC-16ADVP binary pump, DCou-14 A degasser and Shodex PL Hi-PlexPb column (Sc 1011 No. H706081), Guard column Sc-LcShodex, and heater set at 65 °C. The separation and the quantitation were carried out on an amino-bonded column with a mobile phase of CH_3_CN and H_2_O (80/20 V: V) (AOAC, 1997; Ball, 1990; Li, Andrewsw & Pehrssonw, 2002). Standard solutions of Dextran 2,000, 50,000 and 60,000–90,000 with analytical grades were prepared by diluting each concentration in 100 ml deionized water. Injection volume was 20 µl.

#### Estimation of polysaccharide content (Phenol-sulfuric acid method)

Standard curve was prepared to illustrate the relationship between molecular weight and area under the curve using serial dilution of glucose standard solution. Each polysaccharide was diluted (1:1) with de-ionized water. An aliquot of 0.05 ml 80% phenol was added to each tube used for construction of the standard curve or the tested polysaccharide, to a final volume of 2 ml. After mixing, 5 ml H_2_SO_4_ were added, and mixed well on a vortex. The tubes were allowed to stand for 10 min then vortexed again before reading the absorbance at 490 nm (Nielsen, 2010).

#### Polysaccharide activation

Each 10 mg of the prepared capsular polysaccharides were dissolved in 1.0 ml of 0.1M sodium borate buffer pH 9.0 and activated by the addition of 5 µl of CDAP (1-cyano–4–dimethylaminopyridinium-tetrafluoroborate).The mixture was stirred for 5 min and then an aliquot of 1.4 ml of 0.05M HCl was added (Suarez et al., 2008).

#### Polysaccharide conjugation

Bovine serum albumin (20 mg) was dissolved in 1.0 ml of 0.1M NaHCO_3_ buffer pH 8.3 and added to the activated polysaccharide solution, gently stirred for 8 h at room temperature. Then, an aliquot of 1.0 ml 0.1M Tris–HCl buffer pH 8.6 was added to block any remaining activation sites on the unreacted polysaccharide and incubated for 1 h more. Purification of polysaccharide-protein conjugate was done by ultrafiltration according to the method described before (Mcmaster, 2006).

### Immunogenic assay of the conjugate

#### Immunization of mice and antibody responses

Sixty female BALB/c mice were divided into four groups. Each group included 15 mice which were further divided into three subgroups, each containing five mice. Control group (A) received 0.5 ml of 1 mg/ml Alum, group (B) 0.5 ml of 4 µg/ml BSA, group (C) 0.5 ml of 2 µg/ml for each pure polysaccharide containing 1 mg/ml alum while group (D) received 0.5 ml of 2 µg/ml for each conjugated polysaccharide mixed with 1 mg/ml alum. Mice were injected intraperitoneally (I.P.) according to the specified groups; a second round of immunization was performed 14 days after the first one (Meng et al., 2009).

Blood samples were collected by puncturing the retro-orbital plexus vessels of mice at days 0, 14 and 28 of the immunization protocol (Hoff, 2000). Retro-orbital bleeding was done under general anesthesia. Enzyme-linked immunosorbent assay (ELISA) was carried out using 96-well polystyrene microtiter plates activated with 100 µl of poly-L-lysine at 37 °C for 2 h (one plate for each determined polysaccharide). Plates were washed three times with double distilled water and then coated with 100 µl of 10 mg/ml of each prepared polysaccharide diluted in PBS, pH 7.6, at 4 °C overnight. The plates were washed three times with PBS-I buffer (PBS + 0.05% tween 20), and were blocked with 300 µl PBS-II buffer (PBS + 0.05% tween 20 + 1% BSA). at 37 °C for 2 h then washed three times with PBS-I buffer. An aliquot of 100 µl antiserum of each subgroup in the four groups was added to each well and the plate was incubated at 37 °C for 2 h. After three washes with 300 µl of PBS-I, the plates were incubated with 100 µl HRP-labeled goat antimouse antibody for 2 h at room temperature, the plates were then washed three times with PBS-I buffer. An aliquot of 100 µl of the substrate mixture was used to develop the chromogenic reaction and kept in the dark at 20–25 °C for 30 min. The reaction was stopped using 100 µl 0.6 % H_2_SO_4_/ well and absorbance was measured at 490 nm using an ELISA plate reader (Meng et al., 2009).

#### Active protection by immunization with conjugated polysaccharides (challenge test)

Forty female BALB/c mice were divided into four groups (10 mice each). Group I and II received 0.5 ml 1 mg/ml alum, used as control groups, group III received 0.5 ml containing 2 µg/ml 6 A/B conjugated polysaccharide plus 1 mg/ml alum and group IV received 0.5 ml containing 2 µg/ml of 19F conjugated polysaccharide plus 1 mg/ml alum. Mice were injected intraperitoneally according to the specified group; second immunization round was performed 14 days later.

Each immunized BALB/c mouse was then infected with approximately 10^7^ CFU of either the capsular type 6A/B bacteria or the type 19F bacteria. Groups I and III received intraperitoneal injections with the challenge dose of strain 6A/B, with group I used as control. Groups II and IV received intraperitoneal injections with the challenge dose of strain 19F, with group II used as control. Mice were monitored for survival over 21 days. The survival time of each mouse was recorded. Differences in median survival time between groups were analyzed by constructing Kaplan Meier curves. Differences in the overall survival rate between groups were analyzed by the Fisher exact test (Ogunniyi et al., 2000).

### Statistical analysis

Statistical analyses were performed using GraphPad Prism software (version 6.01) (GraphPad Software, Inc., La Jolla, CA, USA). Upon comparing groups, a two-way ANOVA was applied. Mice systemic infection survival experiment was analyzed by applying the log-rank (Mantel–Cox) and Gehan–Breslow–Wilcoxon tests. In all analyses, the *p* values ≤ 0.05 were considered significant.

## Results

### Identification of the most predominant serotypes

In our previous study, a total of 100 clinical specimens were collected in the greater Cairo area in Egypt from 2011 to 2013. Isolates were cultured and identified as *S. pneumoniae*, the serotyping of which revealed that the most common serotypes are 6A/B (30%) followed by 19F (28%), (Table 1) (Bahy et al., 2016). Consequently, the serotypes 6A/B and 19F were considered as the most predominant serotypes.

**Table 1:** Percentages of prevalent serotypes.

Serotype	Percentage
6A/B	30
19F	28
5	15
23F	7
Other	20

### Extraction and purification of capsular polysaccharides

Capsular polysaccharides were isolated from bacterial cells using sodium deoxycholate for lysis and ethanol for precipitation. Further purification from nucleic acid was done using DNase and RNase enzymes, and from proteins using phenol-acetate buffer.

### Polysaccharides identification and polysaccharide content estimation

**Figure 1:**
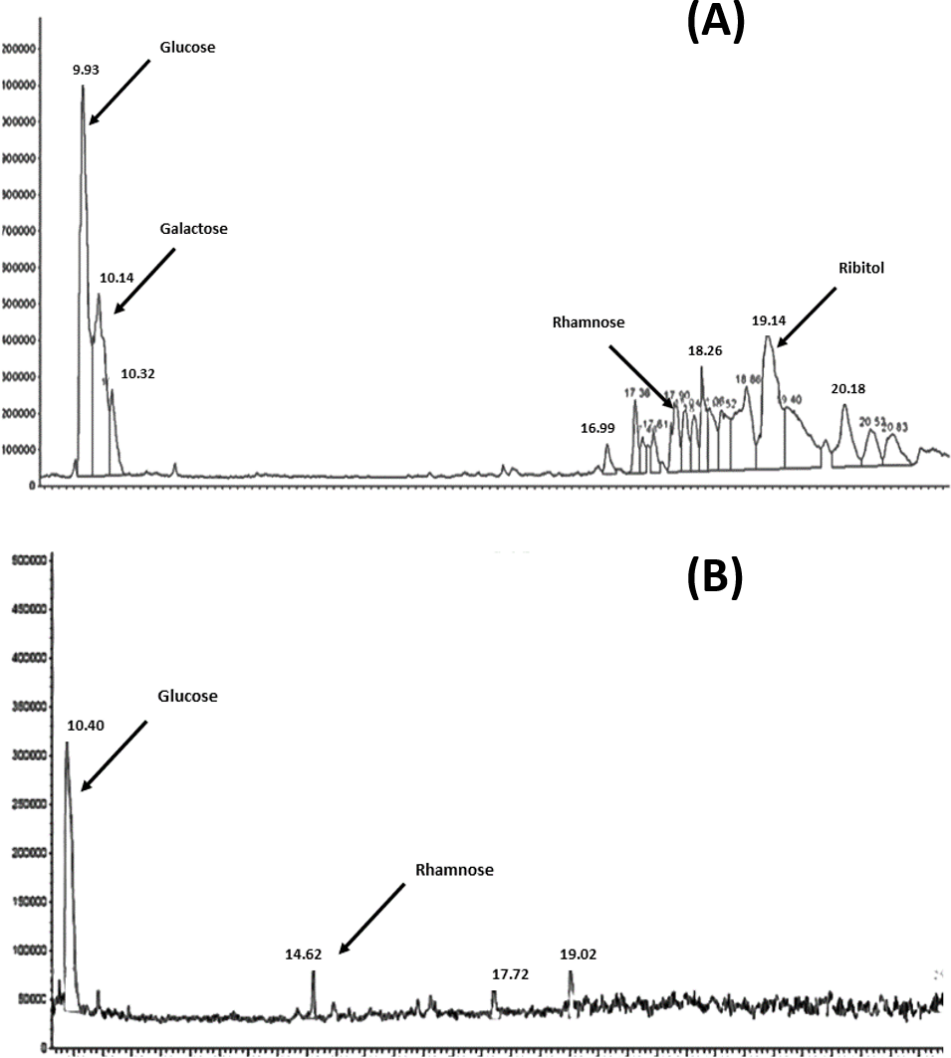
GC–MSD total ion chromatograms for *S. pneumoniae* serotype 6A/B AND 19F at the purified polysaccharides stage. (A) Complete hydrolysis down to the monosaccharide of glucose, galactose, rhamnose and ribitol was observed in case of 6A/B. (B) Hydrolysis was only down to monosaccharides of glucose and rhamnose in case of 19F.

The results of the analysis of the purified polyscharides using gas chromatography is presensted in Figs. 1A and 1B. The resulting chromatograms show the monosaccharide analysis of serotype 6A/B and 19F, the labeled peaks indicate the major methyl glycoside peaks for each monosaccharide. The polysaccharide content estimation using the Phenol-Sulphoric assay showed that it was 83.6 µg/ml for 6A/B, and 13.6 µg/ml for 19F. The difference in the final amounts obtained is attributed to the difference in the chemical structure and monosaccharide composition between the two serotypes.

### Molecular size distribution of 6A/B and 19F purified polysaccharides

**Figure 2:**
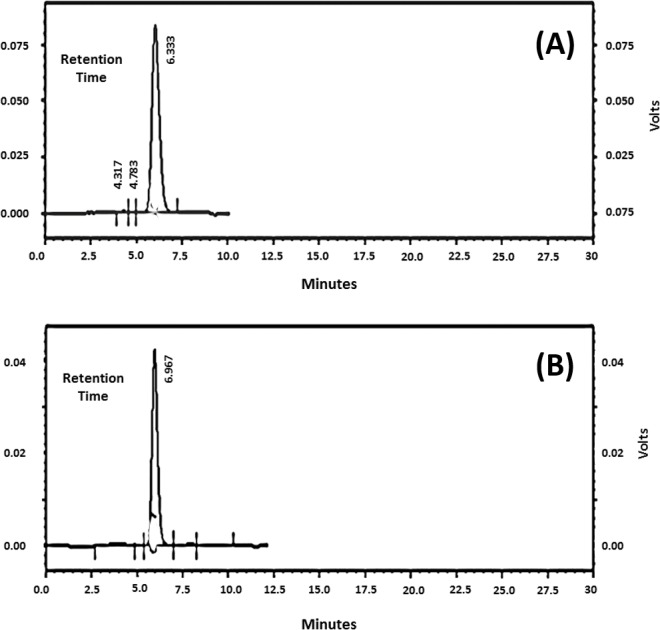
Chromatograms of (A) Polysaccharide of serotype 6A/B with molecular weight of 12, 259 KDa. (B) Polysaccharide of serotype 19F with molecular weight of 30, 343 KDa.

Samples of pneumococcal polysaccharide 6A/B and 19F were analyzed individually and the chromatograms were obtained revealing one main peak for each (Figs. 2A and 2B). The molecular weights (MW) were found to be 12, 259 KDa for 6A/B and 30, 343 KDa for 19F. The commercial vaccine (Synflorix) was used as a control however, although formed of ten serotypes, its chromatogram showed only six peaks instead of ten as several polysaccharides had close molecular weights (not shown). Determination of the polysaccharide and protein content before and after polysaccharide-protein conjugate purification revealed a significant decrease after purification using ultrafiltration with ammonium sulfate solutions, this is probably due to removal of the unbounded polysaccharides and protein by dialysis.

### The purified polysaccharides and polysaccharides conjugates elicit a humoral immune response in mice

**Figure 3:**
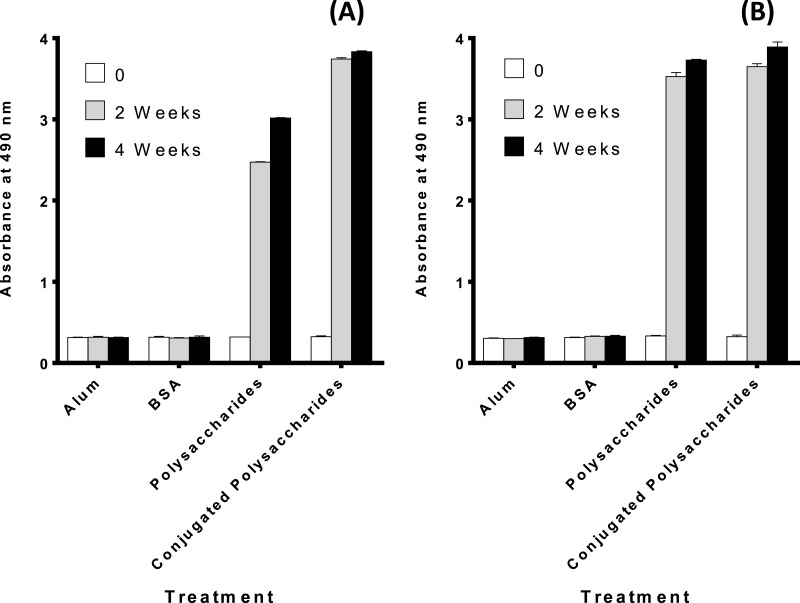
Antibody levels against: (A) serotype 6A/B conjugated pneumococcal polysaccharides, results indicate a highly significance difference in the antibody levels against pure and conjugated 6 A/B pneumococcal polysaccharide in both week 2 and 4 boost immunization, (*p* < 0.0001) for both time intervals. (B) Serotype 19F conjugated polysaccharides, results indicate significant difference in the antibody levels against pure and conjugated 19F pneumococcal polysaccharide in both week 2 and 4 boost immunization, (*p* < 0.0001) for both time intervals.

Figures 3A and 3B demonstrates that both the polysaccharides and the conjugated polysaccharide-protein for both serotypes induced higher immune response than alum or BSA alone (*p* < 0.0001 indicating a significant difference).

### The developed vaccine offer protecion in the penumococaal challenge test

**Figure 4:**
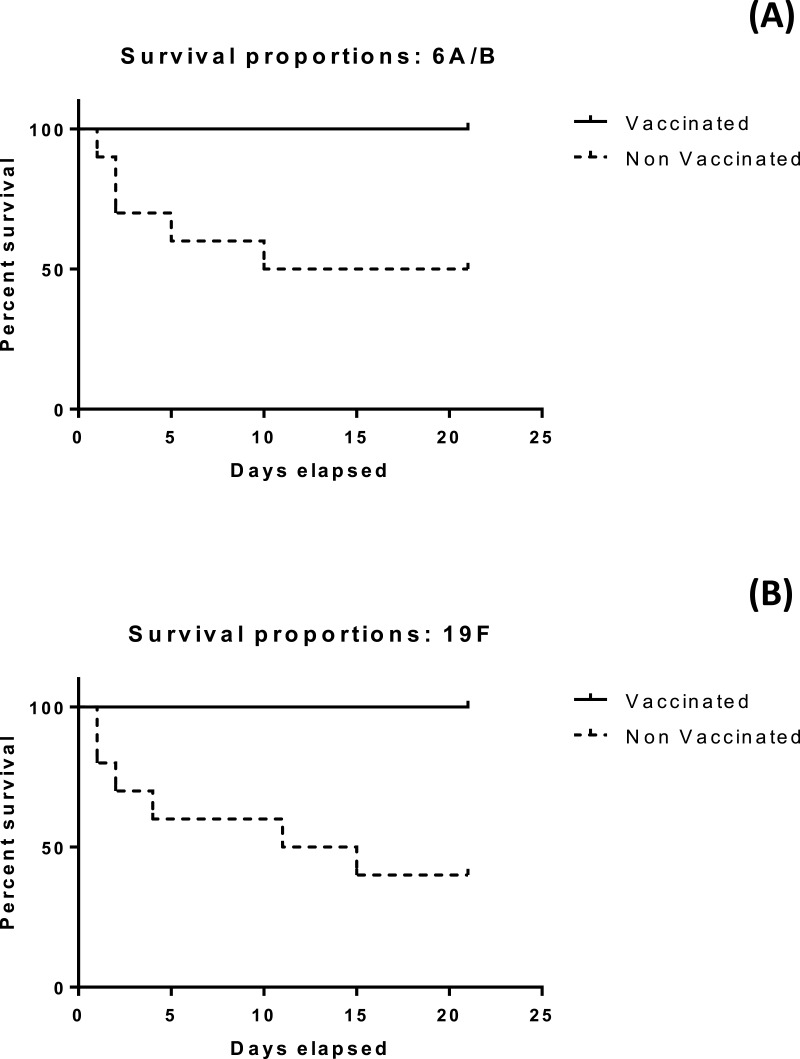
Kaplan Meier survival plots for mice vaccinated with (A): 6A/B, (B): 19F. In both cases, a significant difference is observed between vaccinated and non-vaccinated groups (*p* < 0.05). In case of 6A/B, the *p* value was 0.0115 based on log-rank (Mantel–Cox) test and 0.0124 based on Gehan–Breslow–Wilcoxon test. For 19F, *p* value was 0.0039 based on log-rank (Mantel–Cox) test and 0.0047 based on Gehan–Breslow–Wilcoxon test.

The results of the mice survival over 21 days for the four groups under investigation is illustrated in Figs. 4A and 4B. All mice subjected to vaccination with either serotype survived more than 21 days. Differences in median survival time between groups were analyzed by the Kaplan–Meier survival estimates. For serotype 6A/B conjugate vaccine, the median survival time was 15.5 days for the nonvaccinated group. For serotype 19F conjugate vaccine, the median survival time was 13 days for the nonvaccinated group. Significant difference between the vaccinated and non vaccinated groups was observed in both serotypes at (*p* < 0.05) using log-rank (Mantel–Cox) test (*p* value = 0.0115 and 0.0039 in case of 6A/B and 19F, respectively), and (Gehan–Breslow–Wilcoxon) test (*p* value = 0.0124 and 0.0047 in case of 6A/B and 19F, respectively).

## Discussion

The high cost of importing and the existence of many un-effective serotypes in the imported vaccine make it essential to produce a local vaccine containing only the required most prevalent serotypes. Administration of unnecessary extra serotypes increases the risk of toxicity, lowers the margin of safety and limits the pool of the effective antibodies against the actual prevalent serotypes. The vaccine composition of different serotypes should be updated according to continual epidemiological surveys. Locally produced vaccine will have lower cost, more effectiveness against specific widespread serotypes and with lower risk to receive un-required ones. This study was carried out in order to prepare a conjugate polysaccharide vaccine from the recent predominant serotypes of *S. pneumoniae* prevalent in Egypt which were identified mostly as serotypes 6A/B and 19F accounting together for 58% of the prevalent types.

Prevention of the pneumococcal pneumonia by immunization with specific capsular polysaccharides was first made in the late 1940s (McLeod et al., 1945). In the present study, the capsular polysaccharides of serotype 6A/B and serotype 19F were extracted, purified and used to construct a vaccine as previously described in other studies (Mallick, 2009; Suarez et al., 2001). Gas chromatography (GC) was used as a quantitative tool for the carbohydrate analysis. Excellent resolution and robustness are typically associated with GC applications due to the large number of theoretical chromatographic plates and the inherent purity of the final derivatized sample solubilized into organic solvent. Typical detector used for carbohydrate analysis is the mass selective detector (MSD), which provides additional conformation of the peak assignments by the analysis of mass spectra as well as peak purity.

Anhydrous methanolysis is commonly used to depolymerize polysaccharides into methyl glycosides, which can be subjected to subsequent derivatization and chromatography on a GC–MSD (Merkle & Poppe, 1994; Zanetta, Breckenridge & Vincendon, 1972). Methyl glycosides are relatively stable products compared with monosaccharides released under aqueous conditions and can be subjected to harsher hydrolyses with minimal degradation (Turula et al., 2004; WhitWeld et al., 1991). Pneumococcal polysaccharides were subjected to methanolysis as the sole hydrolysis step, followed by derivatization and chromatography on a GC–MSD. Repeating unit structures for the serotype 6A/B and 19F pneumococcal polysaccharides had already been detected according to (Jennings, Rosell & Carlo, 1980; Kenne, Lindberg & Madden, 1979; Kim et al., 2005; Rebers & Heidelberger, 1961). Serotypes 6A and 6B have the same monosaccharide composition and yield very similar chromatograms, so, monosaccharide composition analysis would not distinguish between them. The gas chromatography charts showed complete hydrolysis down to the monosaccharide of glucose, galactose, rhamnose and ribitol in case of 6A/B and glucose and rhamnose in case of serotype 19F. These results are in agreement with the previous results from serotype 19F polysaccharide hydrolysis (Kim et al., 2005). It was found that serotype 19F does not release its N-acetyl-mannosamine due to linkage to an acid-resistant phosphodiester bond.

Polysaccharide activation by 1-cyano-4-dimethylaminopyridinium-tetrafluoroborate (CDAP) reagent has been optimized and previously applied to protein immobilization. It also has been used for the activation of soluble polysaccharides towards the conjugation with proteins. The use of CDAP has proved to render better yields than the traditional use of cyanogen bromide (CNBr) (Lees, Nelson & Mond, 1996; Suarez et al., 2008). BSA was conjugated to the activated 6A/B and 19F polysaccharides, the effectiveness of these conjugates in vaccination depends on the conjugation and purification method employed in their preparation as it affects the chemical structure and final composition of the product (Suarez et al., 2008).

The immunogenicity of the prepared polysaccharide- protein conjugates was measured using ELISA assay to determine the antibody levels for each immunized group. Control groups had no significant response, while, a highly significant difference in the antibody levels against pure and conjugated 6A/B and 19F pneumococcal polysaccharide was observed in both week 2 and 4 post immunization. However, in the case of serotype 6A/B, it was clear that the increase in conjugated polysaccharide response is high compared to the increase of conjugated polysaccharide in case of serotype 19F, which may be due to the strength of conjugation between polysaccharides and protein in case of 6A/B rather than 19F polysaccharides. The ability of the conjugated vaccine to protect against a lethal challenge with highly pathogenic *S. pneumoniae* 6A/B and 19F in mice was evaluated through active protection and challenge test. Results of the mice survival rate over 21 days for the four groups were recorded. Both the previously vaccinated groups with serotypes 6A/B and 19F survived and were in good health till the end of the experiment time, control unvaccinated groups had different survival time for each mice and only 50% and 60% stayed alive till the end of the experiment respectively. Statistical analysis for the results indicated that there is a significant difference between median survival time of vaccinated groups and unvaccinated groups. Antibody levels elicited by the polysaccharide and the protection ability against pathogenic *S. pneumoniae* serotypes prove its immunogenic capabilities which will still need further clinical studies.

The covalently linked polysaccharide-protein conjugate pneumococcal vaccines have been in use over 10 years and are very successful. The seven-valent conjugate vaccine (Prevenar 7; Wyeth) is approved for use mainly in children younger than two years, and for children younger than five years with high-risk conditions. This vaccine has reduced incidence of invasive pneumococcal disease in children in the USA younger than one year by 82%. One of the most remarkable attributes of the pneumococcal conjugate vaccines has been the degree of herd immunity they generate (Van der Poll & Opal, 2009). Other polysaccharides conjugated vaccines, such as (Synflorix, GSK) and (Prevenar 13, Wyeth), have been used widely all over the world with a remarkable immunogenic and protective effect. However, all the previously mentioned types contain several serotypes which might not be needed in the vaccine. It is essential to formulate vaccines to contain the minimum number of serotypes instead of using the multivalent vaccines with ineffective serotypes and high prices particularly in developing countries.

## Conclusions

We were able to produce a vaccine composed of the most predominant serotypes of S. pneumoniae and to prove its efficiency in an animal model. The prepared conjugated pneumococcal polysaccharides vaccine can serve as a local vaccine specific for vaccination against the most predominant serotypes in Egypt. Future production of this vaccine helps in the control of pneumococcal diseases instead of the importation of the high cost pneumococcal vaccine which may contain other serotypes giving no additional values in diseases control, but may have added side effects.

##  Supplemental Information

10.7717/peerj.2737/supp-1 Data S1 Raw Data for Fig. 3

## Additional Information and Declarations

### Data Availability

The following information was supplied regarding data availability: Raw data can be found in the Supplemental Information.
